# Investigating the Predictive Value of Functional MRI to Appetitive and Aversive Stimuli: A Pattern Classification Approach

**DOI:** 10.1371/journal.pone.0165295

**Published:** 2016-11-21

**Authors:** Ciara McCabe, Vanessa Rocha-Rego

**Affiliations:** 1 School of Psychology and Clinical Language Sciences, University of Reading, Reading, United Kingdom; 2 Instituto de Biofisica Carlos Chagas Filho, University of Rio de Janeiro, Rio de Janeiro, Brazil; Anhui Medical University, CHINA

## Abstract

**Background:**

Dysfunctional neural responses to appetitive and aversive stimuli have been investigated as possible biomarkers for psychiatric disorders. However it is not clear to what degree these are separate processes across the brain or in fact overlapping systems. To help clarify this issue we used Gaussian process classifier (GPC) analysis to examine appetitive and aversive processing in the brain.

**Method:**

25 healthy controls underwent functional MRI whilst seeing pictures and receiving tastes of pleasant and unpleasant food. We applied GPCs to discriminate between the appetitive and aversive sights and tastes using functional activity patterns.

**Results:**

The diagnostic accuracy of the GPC for the accuracy to discriminate appetitive taste from neutral condition was 86.5% (specificity = 81%, sensitivity = 92%, p = 0.001). If a participant experienced neutral taste stimuli the probability of correct classification was 92. The accuracy to discriminate aversive from neutral taste stimuli was 82.5% (specificity = 73%, sensitivity = 92%, p = 0.001) and appetitive from aversive taste stimuli was 73% (specificity = 77%, sensitivity = 69%, p = 0.001). In the sight modality, the accuracy to discriminate appetitive from neutral condition was 88.5% (specificity = 85%, sensitivity = 92%, p = 0.001), to discriminate aversive from neutral sight stimuli was 92% (specificity = 92%, sensitivity = 92%, p = 0.001), and to discriminate aversive from appetitive sight stimuli was 63.5% (specificity = 73%, sensitivity = 54%, p = 0.009).

**Conclusions:**

Our results demonstrate the predictive value of neurofunctional data in discriminating emotional and neutral networks of activity in the healthy human brain. It would be of interest to use pattern recognition techniques and fMRI to examine network dysfunction in the processing of appetitive, aversive and neutral stimuli in psychiatric disorders. Especially where problems with reward and punishment processing have been implicated in the pathophysiology of the disorder.

## Introduction

Distinguishing potentially rewarding and aversive information in the environment is clearly beneficial for self-preservation. Disturbances in the neural systems underlying the processing of positive and negative information are found dysfunctional in psychiatric disorders like depression. In particular it is thought that increased neural processing of negative information underlies and maintains the symptoms of low mood and anxiety in depression [1] whereas the reduced neural response to reward has been proposed as underlying the symptom of anhedonia in depression [2].

Animal studies examining addiction and thus appetitive behaviours have revealed the neural substrates for appetitive responses in regions such as the ventral tegmental area, the nucleus accumbens, and medial prefrontal cortex making up the mesocorticolimbic system [3]. More recently, studies reveal a role in not only reward but also punishment in this system [4]. This focus has been facilitated by the desire to understand better the neurobiological components of dysfunctional responses to reward/appetitive behaviours and aversion in psychiatry and also how current psychological and pharmacological therapies impact upon these systems, if at all [5–9].

Thus far studies in humans suggest that the ventral striatum midbrain and OFC, all rich in dopamine projections respond to appetitive stimuli [10–16] whereas aversive processing seems to engage the amygdala and anterior insula [17–22]. However aversive processing also engages the ventral and dorsal striatum [23–26] while evidence points to amygdala and anterior insula involvement in appetitive processing [27,28]. Thus there seems to be no clear story of functional segregation in brain regions for appetitive and aversive processing. Consistent with this a recent meta analysis examining appetitive and aversive behaviours in animal and human studies reports that overlapping regions in the OFC, striatum, insula and amygdala regions actually respond to both appetitive *and* aversive stimuli and that it is motivation and salience “activations” that are coded in overlapping regions and not only the value of the stimulus *per se* [29].

Further as Hayes et al point out in their meta analysis the direction of the effects found across studies are not consistent due to differences in design and study context for e.g. most studies only report appetitive or aversive processing and not both together. Moreover most studies make it difficult to assess the interactions of region activity to appetitive and aversion as they promote a distinct region by valence hypothesis. Further Hayes et al note how studies differ depending on whether or not the processing of appetitive is active or passive something certainly not consistent across studies. These meta analysis findings question the utility of current fmri methods and thus results in investigating appetitive and aversion processing and instead point to a need to examine how the same systems interact in response to appetitive and aversive stimuli across brain networks.

Thus a more sensitive way to investigate the functional response to stimuli important in the aetiology of depression might be by making use of the recent advances in multivariate pattern recognition techniques. It would help bridge the gap between neuroscience and clinical practice. In this respect, multivariate pattern recognition techniques represent a major development.

The most commonly used pattern recognition algorithm has been the support vector machine (SVM) classifier. The SVM classifier has been used for the classification of patients with Alzheimer’s disease [30,31] autism [32], aphasia [33] and psychosis [34,35] and to predict clinical variables based on patterns of brain activation in functional MRI [36]. However, the SVM classifier yields binary (case or control) and not probabilistic outcomes. For many applications, probabilistic predictions are desirable as they have two key advantages: they provide accurate quantification of predictive uncertainty, reflecting variability within subject groups (e.g. in quantifying the probability that a subject has a psychiatric disorder within a population where illness severity can be expected to vary between individuals), and they allow adjustment of predictions to compensate for different frequencies of diagnostic classes within the general population [36]. GPCs represent a significant advance over SVM as they are fully probabilistic pattern recognition models based on Bayesian probability theory. For neuroimaging, GPCs combine equivalent predictive performance to SVM with the additional benefit of probabilistic classification [37,38].

Therefore, in this study we used GPCs to examine the predictive value of brain network functional data to discriminate appetitive and aversive processing in healthy participants who took part in functional MRI. Given that reward and aversion processing are not unitary constructs but rather have dimensions involving consummatory and anticipatory aspects we examined the brains response during both tastes (consummatory) and sights (anticipation) of reward and aversion. We embedded the classifier with features that correspond to regions known to participate in the processing of appetitive and aversive stimuli [39].

We hypothesised overlapping regions of activity would classify both appetitive and aversive stimuli but that unique patterns of activity would be related to accurately identifying Appetitive vs. Neutral, Aversive vs. Neutral and Appetitive vs. Aversive.

## Material and Methods

### Subjects

Twenty six participants (fifteen females, mean age 25.2; 5,04 SD) were included in this study. Participants were assessed with the Structured Clinical Interview for DSM-IV Axis I Disorders (SCID-I) schedule to exclude a personal current or previous history of major depression or any other axis 1 disorder [40]. All participants were right handed, according to the Edinburgh Handedness Inventory [41] and had normal or corrected to normal vision. Participants with any contraindications for MRI examination or neurological disorders were excluded. Ethical approval was obtained from the Oxford Research Ethics Committee and after complete description of the study to the subjects, written informed consent was obtained. None of the participants took current medication apart from the contraceptive pill.

### Appetitive and aversive stimuli

The stimulus (S1 Table) and experimental design described are the same as published previously [42]. Stimuli were delivered to the subject's mouth through three Teflon tubes (one for the tasteless rinse control described below, one for chocolate taste and one for strawberry taste); the tubes were held between the lips. Each tube was connected to a separate reservoir via a syringe and a one way syringe-activated check valve (Model 14044–5, World Precision Instruments, Inc.), which allowed 0.5 mL of any stimulus to be delivered manually at the time indicated by the computer. The chocolate was formulated to be liquid at room temperature, with a list of the six stimulus conditions described in S1 Table. The aversive stimulus was a medicinal strawberry-flavoured placebo solution (Rosemount Pharmaceuticals Ltd.) which was rated equal in intensity to the chocolate, but unpleasant in valence. A control tasteless solution 0.5 mL of a saliva-like rinse solution (25×10−3mol/L KCl and 2.5× 10−3mol/L NaHCO3 in distilled H2O) was used after every trial that had a taste component, and a control grey image was used after every trial that had a sight only component. Both the liquid chocolate and strawberry had approximately the same texture which enabled them to pass freely through the Teflon delivery tubes.

We included in the GPC analyses the GLM coefficients of the following stimulus: appetitive taste (chocolate in the mouth + gray visual stimulus), neutral taste (tasteless rinse control solution + gray visual stimulus), aversive taste (strawberry in the mouth + gray visual stimulus), appetitive sight (Picture of chocolate), aversive sight (Picture of moldy strawberries), neutral sight (gray visual stimulus).

### Experimental design

At the beginning of each trial, one of the six stimuli chosen by random permutation was presented. If the trial involved an oral stimulus, this was delivered in a 0.5-mL aliquot to the subject's mouth. At the same time, at the start of the trial, a visual stimulus was presented, which was either the picture of chocolate, of moldy strawberries, or a gray control image of approximately the same intensity. The image was turned off after 7 s at which time a small green cross appeared on a visual display to indicate to the subject to swallow what was in the mouth. After a delay of 2 s, the subject was asked to rate each of the stimuli for pleasantness on that trial (with +2 being very pleasant and −2 very unpleasant), for intensity on that trial (0 to +4), and for current wanting for chocolate (+2 for wanting chocolate very much, 0 for neutral, and −2 for very much not wanting chocolate). The ratings were made with a visual analog rating scale in which the subject moved the bar to the appropriate point on the scale using a button box. Each rating period was 5 s long. After the last rating, the gray visual stimulus indicated the delivery of the tasteless control solution that was also used as a rinse between stimuli, and this was administered in exactly the same way as a test stimulus and the subject was cued to swallow after 7 s by the green cross. The tasteless control was always accompanied by the gray visual stimulus. On trials on which only the picture of chocolate was shown, there was no rinse but the gray visual stimulus was shown in order to allow an appropriate contrast as described below. There was then a 2-s delay period similar to other trials that allowed for swallowing followed by a 1-s gap until the start of the next trial. A trial was repeated for each of the six stimulus conditions shown in S1 Table, and the whole cycle was repeated nine times. The instruction given to the subject was (on oral delivery trials) to move the tongue once as soon as a stimulus or tasteless solution was delivered (at the time when the gray visual stimulus was turned on) in order to distribute the solution round the mouth to activate the receptors for taste and smell and then to keep still for the remainder of the 7-s period until the green cross was shown, when the subject could swallow. This procedure has been shown to allow taste effects to be demonstrated clearly with fMRI, using the procedure of subtracting any activation produced by the tasteless control from those produced by a taste or other stimulus [42].

### Functional MRI data acquisition

The experimental protocol consisted of an event-related interleaved design. Images were acquired with a 3.0-T Varian/Siemens whole body scanner at the Oxford Centre for Functional Magnetic Resonance Imaging, where T2*-weighted Echo Planar Imaging (EPI) slices were acquired every 2 s (TR = 2). Imaging parameters were selected to minimize susceptibility and distortion artifact in the orbitofrontal cortex [43]. Coronal slices (25) with in-plane resolution of 3×3 mm and between plane spacing of 4 mm were obtained. The matrix size was 64×64, and the field of view was 192×192 mm. Acquisition was carried out during the task performance yielding 972 volumes in total. A whole-brain T2* weighted EPI volume of the above dimensions, and an anatomical T1 volume with coronal plane slice thickness 3 mm and in-plane resolution of 1.0×1.0 mm was also acquired.

### Preprocessing data

Imaging data were preprocessed and analysed using statistical parametric mapping software SPM5 (http://www.fil.ion.ucl.ac.uk/spm/). Data preprocessing included realignment, normalisation to the Montreal Neurological Institute (MNI) coordinate system, reslicing with sinc interpolation, 6 mm half maximum and spatial smoothing with a full width isotropic Gaussian kernel and global scaling [44]. For each voxel, time series non-sphericity was accounted and corrected for [45], and a low-pass filter was applied (with a haemodynamic response kernel), as was a high-pass filter, with a cut-off period of 128 s. Stimulus onsets were modeled as single impulse response functions and convolved with canonical haemodynamic response function, to which a general linear model was applied to the time course of activation.

### Feature extraction and feature selection

For each subject a general linear model (GLM) was constructed in SPM5 with the neural response to the passive receipt of appetitive, aversive and neutral sight and taste stimuli entered in the design matrix as separate regressors. Movement parameters from the realignment stage were entered as covariates of no interest to control for subject movement. The images corresponding to the GLM coefficients for each experimental condition (appetitive, aversive and neutral) defined the spatial patterns of brain activation used as input to the classifier. In order to select features, based on the recent meta-analyses [29] we include regions known to play a key role in appetitive and aversive processing such as hippocampus, thalamus, amygdala, anterior cingulate cortex, midcingulate cortex, periaqueductal grey, insula, accumbens, putamen, caudate, and regions from orbital, temporal and frontal gyri.

### Pattern Classification Analyses

In this study, we used as classifier the GPC. Technical descriptions of GPC inference have been presented elsewhere [37]. Briefly, the classifier is first trained to determine a set of parameters that best distinguished between cases and controls; the GPC parameters are computed by maximizing the logarithm of the marginal likelihood. In the test phase, the classifier predicts the group membership of a previously unseen example. In order to classify each new example we first multiply each voxel by its correspondent coefficient in the weight vector. We then add all multiplied values and pass the sum through a sigmoid function in order to obtain an output between 0 and 1 (which are predictive probabilities). In this study the GPC was implemented in the PROBID software package (http://www.brainmap.co.uk/PROBID). We examined the ability of the GPC to discriminate between different appetitive and aversive stimuli using functional activity patterns.

### Permutation Test

Statistical significance of the classifier was determined by permutation testing. This test was used to derive a p-value to determine whether classification accuracy exceeded chance levels (50%). To achieve this, we permuted the class labels from the training set 1000 times (i.e., each time randomly assigning class labels to each structural MRI pattern). We then counted the number of times the permuted test accuracy was higher than the one obtained for the true labels. Dividing this number by 1000 we derived a p-value for the classification accuracies.

### GPC Discrimination Maps

An important secondary outcome from GPC is a spatial representation of the decision boundary or discrimination map [36]. The discrimination map is a spatial representation of the vector of GPC predictive weights and describes the relative contribution of each brain voxel to the classifier decision. Since the discrimination is based on the whole pattern, rather than on individual regions, all voxels within the pattern contribute the classification and no local inferences based on these approaches should be made. Technical details of GPC discrimination mapping have been published elsewhere [36,37]. Due to the multivariate character of the GPC, the discrimination maps should not be interpreted as describing focal effects within individual brain regions. Instead they represent a spatially distributed pattern of coefficients that quantify the contribution of each voxel to the GPC 13 decision function (i.e. the value of a voxel in the discrimination map reflects its contribution or predictive value towards one class or the other). We used the following convention: class 1 was the emotional condition with labels +1 and class 2 was the neutral condition, with labels -1. In the comparison between appetitive and aversive conditions, class 1 was the appetitive condition with labels +1 and class 2 was the aversive condition, with labels -1. In the discrimination map, positive coefficients indicate voxels with predictive value for appetitive or aversive (class 1) (visualized in red colour scale) while negative coefficients indicate voxels with predictive value for neutral (class 2) (visualized in blue colour scale). Comparing appetitive with aversive conditions, positive coefficients indicate voxels with predictive value for appetitive (class 1) (visualized in red colour scale) while negative coefficients indicate voxels with predictive value for aversive (class 2) (visualized in blue colour scale).

## Results

**Fig 1:**
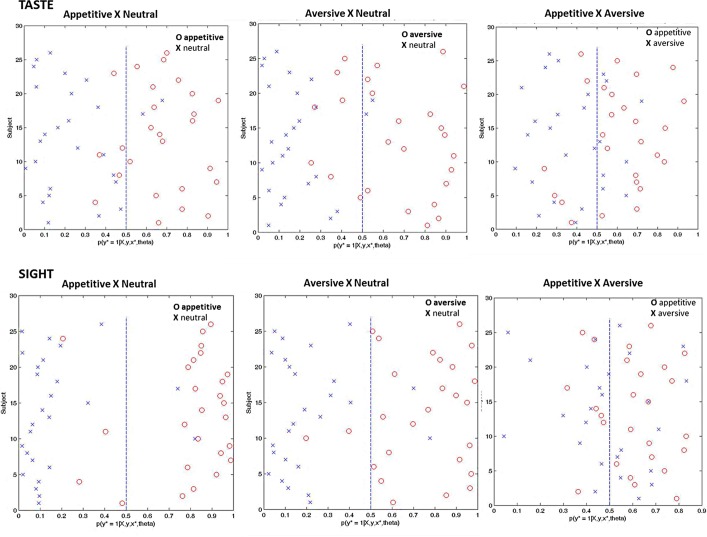
Classification probabilities for positive and negative classifiers.

Fig 1 and Table 1 summarizes the results of the classification between emotional conditions utilizing neural activity patterns elicited to the passive receipt of appetitive, aversive and neutral stimuli from sight and taste modalities. Classification accuracy reflects the predictive power of the algorithm, for taste modality, the accuracy to discriminate appetitive from neutral condition was 86,5% (specificity = 81%, sensitivity = 92%, p = 0.001). In other words, based on a functional imaging, if a participant experienced appetitive taste stimuli the probability of correct classification was 81%. Conversely, if a participant experienced neutral taste stimuli the probability of correct classification was 92%. The accuracy to discriminate aversive from neutral taste stimuli was 82,5% (specificity = 73%, sensitivity = 92%, p = 0.001) and appetitive from aversive taste stimuli was 73% (specificity = 77%, sensitivity = 69%, p = 0.001). In the sight modality, the accuracy to discriminate appetitive from neutral condition was 88,5% (specificity = 85%, sensitivity = 92%, p = 0.001), to discriminate aversive from neutral taste stimuli was 92% (specificity = 92%, sensitivity = 92%, p = 0.001), and to discriminate aversive from appetitive taste stimuli was 63,5% (specificity = 73%, sensitivity = 54%, p = 0.009).

**Table 1:** GPC results of the classification between different emotional conditions.

Taste	Sensitivity	Specificity	Accuracy	p value
Appetitive X Neutral	81%	92%	86,5%	= 0.001
Aversive X Neutral	73%	92%	82,5%	= 0.001
Appetitive X Aversive	77%	69%	73%	= 0.001
Sight				
Appetitive X Neutral	85%	92%	88,5%	= 0.001
Aversive X Neutral	92%	92%	92%	= 0.001
Appetitive X Aversive	73%	54%	63,5%	= 0.009

### Discrimination maps

The discrimination maps highlight a set of regions, which according to our classification approach carry the most distinctive characteristics between the appetitive versus neutral, aversive versus neutral and appetitive versus aversive stimuli in sight and taste modalities. We selected the peaks of the GPC weight vector for each classifier, setting the threshold value to 30% of the maximum (absolute) weight value, and estimated the anatomical regions (cluster peaks) that most contributed to the classifier in the discrimination between the conditions.

#### Appetitive x Neutral

The highest discriminative regions in pleasant and neutral classification to both modalities were localized in superior frontal gyrus, inferior frontal gyrus, superior frontal sulcus, lateral orbital gyrus, precentral gyrus, pregenual anterior cingulate cortex, anterior middle cingulate cortex, posterior middle cingulate, insula and amygdala.

#### Aversive x Neutral

The cluster with the highest discriminative weights in unpleasant and neutral classification to both modalities were superior frontal gyrus, inferior frontal gyrus, middle frontal gyrus, pregenual anterior cingulate cortex, middle cingulate cortex, posterior middle cingulate, superior temporal gyrus, insula, amygdala and hippocampus.

#### Appetitive x Aversive

The highest discriminative regions in pleasant and unpleasant classification to both modalities were localized in superior frontal gyrus, inferior frontal gyrus, middle frontal gyrus, superior frontal sulcus, posterior orbital gyrus, anterior middle cingulate cortex, posterior middle cingulate, superior temporal gyrus, insula, amygdala and thalamus.

**Fig 2:**
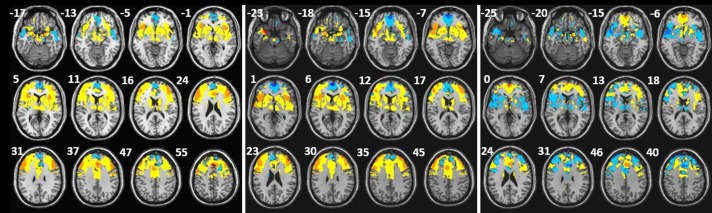
2A. Discrimination maps for appetitive versus neutral emotional taste condition classification, the color code shows the relative weight of each voxel for the decision boundary (red scales: higher weights for neutral and blue scales: higher weights for appetitive; x, y, z, are in MNI coordinates). 2B. Discrimination maps for aversive versus neutral emotional taste condition classification (red scales: higher weights for neutral and blue scales: higher weights for aversive; x, y, z, are in MNI coordinates). 2C. Discrimination maps for appetitive versus aversive emotional taste condition classification (red scales: higher weights for aversive and blue scales: higher weights for appetitive; x, y, z, are in MNI coordinates). The z-coordinate for each axial slice in MNI space is given in millimeters.

**Fig 3:**
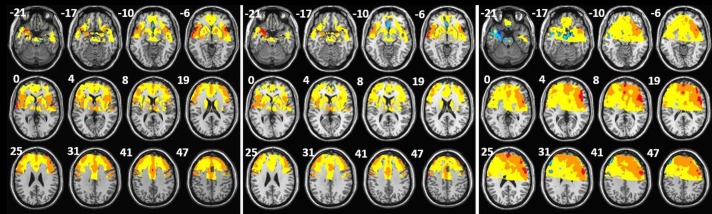
3A. Discrimination maps for appetitive versus neutral emotional sight condition classification, (red scales: higher weights for neutral and blue scales: higher weights for appetitive; x, y, z, are in MNI coordinates). 3B. Discrimination maps for aversive versus neutral emotional sight condition classification, (red scales: higher weights for neutral and blue scales: higher weights for aversive; x, y, z, are in MNI coordinates). 3C. Discrimination maps for appetitive versus aversive emotional sight condition classification, (red scales: higher weights for aversive and blue scales: higher weights for appetitive; x, y, z, are in MNI coordinates). The z-coordinate for each axial slice in MNI space is given in millimeters.

Discrimination maps showing the global spatial pattern by which emotional taste conditions differ are illustrated in Fig 2 for appetitive versus neutral, for aversive versus neutral and for appetitive versus aversive and detailed in Tables 2, 3 and 4. The brain regions that contribute to discriminate the emotional sight conditions are illustrated in Fig 3 for appetitive versus neutral, for aversive versus neutral and for appetitive versus aversive and detailed in S2–S4 Tables. Some regions showed discriminative value to all classifications superior frontal gyrus, inferior frontal gyrus, middle frontal gyrus, pregenual anterior cingulate cortex (ACC), middle ACC, superior temporal gyrus, putamen, caudate, thalamus, amygdala and insula.

**Table 2:** Regions discriminating between appetitive versus neutral emotional taste classification. Coordinates are shown in MNI, Wi: Highest weights within individual clusters.

Region	Laterality	Coordinates			Wi
		x	y	z	
**frontal lobe**	L	-4	53	21	-3.32
superior frontal gyrus	L	-4	25	47	19.94
	R	2	25	47	18.16
	R	18	23	55	-5.78
	L	-10	31	55	3.41
inferior frontal gyrus	L	-50	31	21	11.54
	L	-52	21	29	11.97
	R	46	27	29	12.78
	L	-42	51	5	7.96
	R	42	43	5	11.62
middle frontal gyrus	L	-36	45	29	5.10
	R	30	49	29	5.43
superior frontal sulcus	L	-20	7	55	3.28
	R	-2	59	7	-9.27
medial orbital gyrus	R	34	19	-13	5.4
	L	-34	15	-3	4.02
	L	-18	33	-17	2.5
	R	18	29	-17	9.26
lateral orbital gyrus	L	-36	23	-5	13.86
	R	44	17	-5	10.13
pregenual anterior cingulate cortex	R	4	49	-11	-3.28
	R	2	59	5	-10.7
	L	-2	59	7	-9.27
subgenual anterior cingulate cortex	R	2	31	-13	-1.8
anterior middle cingulate cortex	L	-6	23	21	10.45
	R	2	25	21	10.36
posterior middle cingulate cortex	R	8	1	31	3.3
	L	-6	-1	31	3.35
**temporal lobe**					
superior temporal gyrus	L	-46	11	-21	-3.37
**parietal lobe**					
insula	R	46	7	-13	-2.65
	L	-36	23	5	11.13
	R	34	17	5	11.59
**amygdala**	L	-20	-3	-21	13.8
**accumbens**	L	-8	7	-11	4.80
	R	10	7	-11	2.60
**putamen**	L	-24	5	-5	4.66
	R	22	9	-5	6.02
**caudate**	L	-6	7	-5	7.78
	R	8	9	-5	7.5
**thalamus**	L	-2	19	21	5.34
	L	-6	-17	1	6.75
	R	6	-17	1	4.48
	L	-6	-17	5	6.36

**Table 3:** Regions discriminating between aversive versus neutral emotional taste classification. Coordinates are shown in MNI, Wi: Highest weights within individual clusters.

Region	Laterality	Coordinates			Wi
		x	y	z	
**frontal lobe**					
superior frontal gyrus	R	4	47	-13	-8.35
	L	-2	49	-12	-10.34
	L	-4	53	17	-3.4
	R	2	55	17	-5.06
inferior frontal gyrus	L	-44	43	7	5.74
	R	44	45	7	5.82
	L	-44	39	17	9.6
	R	44	37	17	10.27
middle frontal gyrus	R	46	41	21	14.63
	L	-46	37	21	9.8
superior frontal sulcus	R	20	19	51	-3.8
	L	-22	27	51	-3.23
pregenual anterior cingulate cortex	R	2	59	-1	-17.02
	L	-4	57	1	-14.97
subgenual anterior cingulate cortex	R	4	33	-13	-3.15
	R	2	31	-11	-3
anterior middle cingulate cortex	R	4	13	24	1.94
	L	-2	25	17	-1.2
posterior middle cingulate cortex	L	-2	3	29	2.78
	R	2	1	29	2.93
**temporal lobe**					
superior temporal gyrus	L	-60	-9	-3	8.37
	R	56	-7	-3	4.77
middle temporal gyrus	R	58	-15	-19	-3.34
**parietal lobe**					
insula	L	-44	15	-3	11.03
	R	44	17	-17	11.66
**amygdala**	L	-26	3	-23	20.32
** **	R	26	1	-19	8.43
**putamen**	L	-20	9	3	6.87
	R	22	7	-3	3.96
**caudate**	R	16	9	-9	3.78
	L	-10	9	-3	7.39
**thalamus**	L	-10	-9	9	7.61
	R	6	-9	7	6.97
**parahipopocampal gyrus**	R	32	-21	-19	-2.29
**hippocampus**	L	-28	-15	-23	2.94

**Table 4:** Regions discriminating between for appetitive versus aversive emotional taste classification. Coordinates are shown in MNI, Wi: Highest weights within individual clusters.

Region	Laterality	Coordinates			Wi
		x	y	z	
**frontal lobe**					
superior frontal gyrus	L	-2	53	-9	14.23
	L	-2	55	-3	17.01
	R	6	53	-9	13.71
	R	2	57	-3	18.89
	L	-2	49	41	-7.64
	R	8	51	41	-4.43
	R	22	13	57	24.14
inferior frontal gyrus	L	-56	11	3	-12.34
	L	-52	25	19	6.06
	L	-50	9	19	-5.27
	R	50	11	3	-4.65
	R	52	21	19	5.33
middle frontal gyrus	L	-40	51	25	-11.94
	L	-28	59	19	-4.42
	R	38	53	3	10.16
	R	32	47	19	-2.28
inferior frontal sulcus	L	-36	47	19	-3.28
	R	38	43	19	-2.36
superior frontal sulcus	L	-24	35	31	-3.34
	R	26	27	31	-3.41
	L	-20	31	41	8.01
	R	24	23	41	2.08
	R	16	19	45	17.57
	L	-22	21	45	4.57
medial orbital gyrus	L	-12	51	-17	8.31
	R	14	47	17	4.00
posterior orbital gyrus	L	-28	27	-17	-5.13
	L	-38	27	-13	-10.08
	R	28	19	-13	-3.15
pregenual anterior cingulate cortex	R	2	45	-3	3.96
subgenual anterior cingulate cortex	L	-2	25	-13	2.54
anterior middle cingulate cortex	R	2	23	27	-11.83
	R	2	23	21	16.22
	L	-2	29	13	-14.23
	L	-2	25	21	20.73
posterior middle cingulate cortex	L	-2	-1	29	-3.25
	L	-2	-13	37	9.54
	R	2	-11	31	-3.29
	R	2	-11	43	3.31
inferior precentral sulcus	R	46	-9	31	3.7
	L	-38	-1	53	5.7
**temporal lobe**					
superior temporal gyrus	L	-38	13	-25	-6.38
	R	44	3	-21	-4.45
	L	-44	3	-17	-11.58
superior temporal sulcus	L	-48	3	-25	-4.1
	L	-52	-3	-21	-5.03
inferior temporal gyrus	R	58	-17	-21	2.79
**parietal lobe**					
insula	L	-44	5	-13	-14.6
	L	-48	9	-9	-15
	R	48	1	-9	-6.6
	R	40	-1	-13	-8.80
**amygdala**	L	-26	1	-25	-15.74
	R	28	-3	-21	-5.65
	L	-24	1	-17	-11.14
**accumbens**	L	-6	7	-11	3.44
	R	4	11	-11	2.97
**putamen**	L	-14	-9	-9	-3.29
**hypothalamus**	R	2	-1	-9	2.18
	L	-4	-3	-9	3.21
**caudate**	L	-14	15	3	3.0
	R	14	21	3	-2.58
	L	-16	-5	19	2.82
	R	10	-3	19	4.35
**thalamus**	L	-2	-17	9	-9.39

## Discussion

The results of our analysis suggest that brain responses to appetitive, aversive and neutral stimuli are associated with distinct patterns of activity and co-activation across the brain. This study evaluated the feasibility of using pattern recognition algorithms for the automatic classification of emotional and neutral taste and sight fMRI responses in healthy controls. The brain patterns can thus diagnose which condition is being presented with accuracy 63.5%% to 92%.

Classification accuracy reflects the predictive power of the algorithm to discriminate different stimulus. For example we found that classification accuracy using GPC analysis of the neural activity patterns elicited to appetitive and neutral stimuli from taste modalities was 86,5%. In other words, if a participant was exposed to an appetitive stimulus, the probability of correct classification was 81%. Conversely, if a participant were exposed to neutral stimulus, the probability of correct classification was 92%. In all comparisons between emotional and neutral stimuli the best probability of correct classification was obtained to the neutral stimulus (see Table 1). In summary, our results show that appetitive and aversive stimuli were better discriminated when compared to neutral stimuli than compared with each other. We speculate that the healthy subjects present less variability in the pattern of brain activation of neutral stimuli compared to emotional stimuli. This could explain the higher accuracy to discriminate neutral stimuli from emotional. Our findings parallel other neuroimaging reports that indicate that the best discrimination was obtained when the comparison involved neutral stimuli. Previous studies have shown that predictive probabilities to patterns of brain activation to neutral faces in patients are significantly lower in comparison to the healthy controls. This difference was specific to neutral faces [46]. Another study showed that the best discrimination between at risk and low-risk adolescents was found to be neutral faces [47].

The results indicate that appetitive and aversive conditions are not represented in any one system but across multiple overlapping networks including the superior frontal gyrus, inferior frontal gyrus, middle frontal gyrus, pregenual ACC, middle ACC, superior temporal gyrus, putamen, caudate, thalamus, amygdala and insula. Neuroimaging studies using conventional univariate analyses have established these same brain regions involved in the processing of reward and aversion in the human brain [48] and also in the dysfunctional responses to reward and aversion in depression [48–52]. However, these findings have had limited translational application primarily for three reasons: (a) there is considerable between-group overlap in brain responses derived from group-level neuroimaging analyses [29,53] (b) voxel-based analysis methods are significantly biased toward detecting group differences that are highly localized in space but are limited in detecting group differences that are spatially distributed and subtle [54] and (c) voxel-based analyses do not lend themselves to making predictions at the level of individual subjects.

Our results also indicated regions such as the lateral OFC and posterior cingulate gyrus are involved specifically in the classification of appetitive vs. neutral stimuli which is interesting given previous studies with univariate analysis finding that the lateral OFC responds to aversive stimuli such as unpleasant tastes [15] losing money [55]and disgust [56]. We also found that the hippocampus and a part of the medial cingulate cortex classified specifically the aversion over the neutral and that the thalamus and the posterior OFC specifically classified the appetitive vs. aversive stimuli. These results, therefore, do not at first glance map directly onto traditional views of appetitive and aversion processing occurring in segregated regions [57,58] rather our results are consistent with the more recent data showing that instead of specific brain regions underpinning one emotion vs. another [59] there are overlapping networks, which is indeed consistent with the architecture of the brain[60,61]. Thus a multi-system view of network based activations to both appetitive and aversive stimuli seems more apt. The further benefit of multivariate analysis however is that it can detect different patterns of activity within the *same* networks allowing for a more sensitive approach that can unpick the neural signature related to one emotion/valance vs. another and does not rely simply on the more or less activity approach of univariate fMRI analysis techniques.

The data presented here demonstrate that the inconsistencies and limitations of univariate fMRI analysis may be surmounted with the aid of multivariate pattern recognition techniques. The application of GPC analysis to functional responses to emotional and neutral conditions provided accuracy in the range 63,5%–92%. These results are especially important if we believe that the neurobiological signature related to appetitive stimuli is related to the clinical symptom of anhedonia in depression [62,63] and that the neurobiological signature of aversion processing is related to the clinical symptom of low mood and enhanced negative information processing in depression [64]. Thus where univariate fMRI analysis has failed to decipher these signatures [29] multivariate GPC can provide more biologically meaningful descriptors which in turn may be used help classify, with a brain based approach, depression and its subtypes.

## Supporting Information

S1 Table A list of the stimulus conditions. (DOCX)

S2 Table Regions discriminating between appetitive versus neutral emotional sight classification. Coordinates are shown in MNI, Wi: Highest weights within individual clusters.
(DOCX)

S3 Table Regions discriminating between aversive versus neutral emotional sight classification. Coordinates are shown in MNI, Wi: Highest weights within individual clusters.
(DOCX)

S4 Table Regions discriminating between for appetitive versus aversive emotional sight classification. Coordinates are shown in MNI, Wi: Highest weights within individual clusters.
(DOCX)
