# Supplementary material for: Investigating the Predictive Value of Functional MRI to Appetitive and Aversive Stimuli: A Pattern Classification Approach
Source: PLoS One. 2016 Nov 21;11(11):e0165295. doi: 10.1371/journal.pone.0165295 (PMC5117589; doi:10.1371/journal.pone.0165295)
Supplement: S1 Table — (DOCX) [file pone.0165295.s001.docx]

Supplementary doc for

Title:

**Investigating the predictive value of functional MRI to appetitive and aversive stimuli: A pattern classification approach.**

Ciara McCabe^1^ and Vanessa Rocha-Rego^2^

^1^School of Psychology and Clinical Language Sciences,

University of Reading, Reading, UK.

^2^Instituto de Biofisica Carlos Chagas Filho, University of Rio de Janeiro,

Rio de Janeiro, Brazil.

Corresponding author:

Ciara McCabe

School of Psychology and Clinical Language Sciences,

University of Reading, Reading, RG6 6AL, UK.

Tel:+441183785450 Email: C.McCabe@Reading.ac.uk

| **A list of the stimulus conditions** | |
| --- | --- |
| Condition 1 | Chocolate in the mouth + gray visual stimulus |
| Condition 2 | Picture of chocolate |
| Condition 3 | Chocolate in the mouth + a picture of chocolate |
| Condition 4 | Strawberry in the mouth + gray visual stimulus |
| Condition 5 | Picture of moldy strawberries |
| Condition 6 | Strawberry in the mouth + a picture of moldy strawberries |
| Rinse condition | Tasteless rinse control solution + gray visual stimulus |

Table S2: Regions discriminating between **appetitive** versus **neutral** emotional **sight** classification. Coordinates are shown in MNI, Wi: Highest weights within individual clusters.

| **Region** | **Laterality** | **Coordinates** | | | **Wi** |
| --- | --- | --- | --- | --- | --- |
|  |  | **x** | **y** | **z** |  |
| **frontal lobe** | L | -4 | 47 | -15 | 2.73 |
| superior frontal gyrus | R | 2 | 49 | -15 | 2.25 |
|  | L | -2 | 55 | 23 | 6.54 |
|  | L | -2 | 47 | 47 | 10.04 |
|  | L | -4 | -5 | 65 | 10.29 |
|  | R | 4 | -7 | 65 | 4.49 |
|  | R | 2 | 45 | 23 | 6.15 |
| inferior frontal sulcus | L | -34 | 55 | 15 | 5.36 |
|  | R | 46 | 33 | 23 | 9.52 |
|  | L | -48 | 33 | 23 | 4.71 |
|  | R | 30 | 53 | 15 | 5.95 |
|  | L | -30 | -11 | 65 | 2.06 |
|  | R | 22 | 5 | 65 | 5.78 |
| inferior frontal gyrus | R | 56 | 15 | 7 | 7.08 |
|  | R | 50 | 9 | 7 | 7.83 |
|  | L | -40 | 39 | 15 | 6.68 |
|  | R | 42 | 37 | 15 | 6.34 |
| middle frontal gyrus | R | 36 | 57 | 7 | 5.51 |
|  | L | -34 | 51 | 7 | 2.95 |
|  | L | -32 | 51 | 23 | 5.51 |
|  | R | 38 | 43 | 23 | 7.29 |
|  | L | -32 | 43 | 29 | 4.87 |
| precentral gyrus | L | -58 | -7 | 33 | 11.44 |
|  | R | 54 | -1 | 33 | 9.7 |
| inferior precentral sulcus | L | -52 | -3 | 33 | 6.60 |
|  | L | -46 | 9 | 47 | 6.79 |
|  | R | 42 | 11 | 47 | 6.26 |
| lateral orbital gyrus | R | 46 | 41 | 7 | 6.00 |
| posterior orbital gyrus | L | -48 | 31 | 7 | 6.27 |
| subgenual ACC | L | -1 | 23 | -9 | -2 |
|  | R | 2 | 25 | -11 | 1.5 |
| pregenual ACC | R | 2 | 35 | 3 | 5.52 |
|  | L | -2 | 37 | 5 | 5.16 |
| posterior middle cingulate cortex | R | 2 | -11 | 41 | 6.07 |
|  | R | 2 | 13 | 31 | 2.55 |
|  | L | -2 | -11 | 29 | 1.71 |
|  | L | -2 | -11 | 45 | 9.5 |
|  | R | 1 | -5 | 47 | 9.7 |
|  | L | -2 | -11 | 37 | 6.38 |
| anterior middle cingulate cortex | R | 4 | 17 | 29 | 5.16 |
|  | L | -2 | 13 | 29 | 4.9 |
|  | L | -2 | 11 | 37 | 9.6 |
|  | R | 1 | 11 | 37 | 9.5 |
| **temporal lobe** |  |  |  |  |  |
| superior temporal gyrus | L | -40 | 11 | -22 | 5.05 |
|  | R | 50 | 5 | -15 | 2.9 |
|  | L | -50 | -5 | -13 | 2.15 |
|  | L | -56 | 3 | -5 | 12.3 |
| middle temporal gyrus | R | 48 | -9 | -23 | 2.20 |
| **parietal lobe** |  |  |  |  |  |
| insula | R | 34 | 9 | -15 | 6.29 |
|  | L | -42 | 3 | -15 | 7.54 |
|  | R | 46 | 15 | -5 | 11.38 |
|  | L | -44 | 5 | -5 | 8.14 |
| **putamen** | R | 16 | 9 | -5 | 6.60 |
|  | L | -24 | 7 | -5 | 5.0 |
|  | L | -32 | -11 | 7 | 3.4 |
|  | R | 20 | -1 | 7 | 4.52 |
| **caudate** | R | 8 | 13 | -5 | 3.73 |
|  | L | -10 | 13 | -5 | 3.24 |
|  | L | -10 | 3 | 7 | 6.7 |
|  | R | 10 | 3 | 7 | 6.75 |
| **amygdala** | R | 22 | -5 | -19 | 3.43 |
|  | L | -20 | 3 | -21 | 9.3 |
| **thalamus** | R | -10 | -17 | 7 | 7.47 |
|  | L | 8 | -23 | 7 | 6.70 |

Table S3: Regions discriminating between **aversive** versus **neutral** emotional **sight** classification. Coordinates are shown in MNI, Wi: Highest weights within individual clusters.

| **Region** | **Laterality** | **Coordinates** | | | **Wi** |
| --- | --- | --- | --- | --- | --- |
|  |  | **x** | **y** | **z** |  |
| **frontal lobe** |  |  |  |  |  |
| superior frontal gyrus | L | -2 | 47 | 47 | 11.69 |
|  | R | 2 | 47 | 47 | 11.55 |
|  | L | -14 | 59 | 21 | -1.97 |
| inferior frontal sulcus | L | -42 | 43 | 21 | 7.49 |
|  | R | 42 | 41 | 21 | 7.28 |
| inferior frontal gyrus | L | -48 | 23 | 21 | 8.35 |
|  | R | 46 | 25 | 21 | 6.45 |
|  | R | 52 | 7 | 9 | 6.2 |
|  | L | -56 | 11 | 9 | 7.52 |
| middle frontal gyrus | R | 46 | 33 | 27 | 11.71 |
|  | L | -36 | 47 | 27 | 6.13 |
|  | L | -26 | 55 | 15 | 5.2 |
|  | R | 30 | 53 | 15 | 6.52 |
| superior frontal sulcus | L | -22 | 11 | 47 | 2.22 |
|  | R | 24 | 9 | 47 | 3.12 |
| medial orbital gyrus | R | 16 | 33 | -19 | 1.6 |
| posterior orbital gyrus | L | -38 | 13 | -15 | 4.47 |
|  | R | 32 | 11 | -15 | 4.52 |
| subgenual ACC | L | -2 | 29 | -9 | -2.09 |
| pregenual ACC | R | 2 | 49 | 1 | 3.31 |
|  | L | -4 | 47 | 3 | 2.9 |
| middle cingulate cortex | L | -2 | 9 | 39 | 11.97 |
|  | R | 2 | 9 | 39 | 10.44 |
| **temporal lobe** |  |  |  |  |  |
| superior temporal gyrus | L | -60 | -7 | -5 | 16.16 |
|  | R | 58 | -5 | -5 | 6.66 |
|  | R | 56 | -15 | 5 | 7.30 |
|  | L | -42 | 13 | -23 | 1.5 |
| inferior precentral sulcus | L | -50 | 7 | 27 | 6.42 |
|  | R | 52 | 7 | 27 | 6.65 |
| **parietal lobe** |  |  |  |  |  |
| insula | L | -44 | 5 | -5 | 9.25 |
|  | R | 46 | 15 | -5 | 12.16 |
| **putamen** | L | -22 | 7 | -5 | 6.19 |
|  | R | 16 | 9 | -5 | 6.48 |
| **caudate** | L | -10 | 13 | 7 | 3.44 |
|  | R | 12 | 13 | 7 | 3.11 |
| **thalamus** | L | -4 | -19 | -15 | 3.59 |
|  | R | 6 | -17 | -15 | 3.15 |
| **amygdala** | L | -22 | 3 | -23 | 17.12 |
|  | R | 20 | -3 | -19 | 7.46 |
| **hippocampus** | L | -18 | 11 | -23 | 9.22 |
| **globo pallidus** | L | -8 | -13 | 7 | 7.12 |
|  | R | 2 | -19 | 7 | 6.9 |

Table S4: Regions discriminating between for **appetitive** versus **aversive** emotional **sight** classification. Coordinates are shown in MNI, Wi: Highest weights within individual clusters.

| **Region** | **Laterality** | **Coordinates** | | | **Wi** |
| --- | --- | --- | --- | --- | --- |
|  |  | **x** | **y** | **z** |  |
| **frontal lobe** | L | -2 | 43 | 3 | 4.9 |
| superior frontal gyrus | R | 8 | 47 | 3 | 4.22 |
|  | L | -16 | 55 | 23 | 5.12 |
|  | R | 24 | 49 | 23 | 4.9 |
|  | L | -10 | -9 | 53 | 8.65 |
|  | R | 10 | 35 | -11 | 3.75 |
|  | L | -2 | 41 | -11 | 4.18 |
| inferior frontal gyrus | R | 42 | 31 | 23 | 7.73 |
|  | R | 44 | 37 | 17 | 7.91 |
|  | L | -38 | 39 | 17 | 2.88 |
|  | R | 46 | 19 | 17 | 7.43 |
|  | L | -52 | 15 | 17 | 2.6 |
| middle frontal gyrus | R | 36 | 47 | 23 | 5.66 |
|  | L | -34 | 37 | 23 | 1.8 |
|  | L | -44 | 37 | 33 | -1.35 |
|  | R | 38 | 43 | 33 | -2.29 |
|  | L | -40 | 43 | 3 | 4.69 |
|  | R | 14 | 21 | 53 | 8.65 |
|  | L | -12 | 25 | 53 | 5.25 |
| inferior precentral sulcus | R | 54 | 5 | 23 | 9.25 |
| superior frontal sulcus | L | -30 | -3 | 57 | -1.37 |
|  | L | -22 | 17 | 53 | 3.86 |
| medial orbital gyrus | L | -6 | 35 | -21 | 4.03 |
|  | L | -6 | 33 | -21 | 3.62 |
|  | L | -4 | 35 | -17 | 3.06 |
|  | L | -18 | 31 | -17 | 4.93 |
| lateral orbital gyrus | L | -40 | 23 | -5 | 4.16 |
| posterior orbital gyrus | R | 42 | 13 | -11 | 7.30 |
| precentral gyrus | L | -54 | 31 | 7 | 4.15 |
|  | L | -54 | -5 | 23 | 4.54 |
|  | R | 52 | 1 | 33 | 9.40 |
| superior precentral sulcus | L | -42 | -1 | 53 | 2.56 |
|  | R | 40 | -1 | 53 | 5.6 |
| poscentral gyrus | R | 54 | -13 | 33 | 3.67 |
|  | L | -38 | -11 | 33 | 1.45 |
| pregenual anterior cingulate cortex | L | -4 | 35 | 13 | 7.9 |
|  | L | -4 | 57 | 21 | 7.8 |
|  | R | 2 | 35 | 13 | 8.58 |
|  | R | 2 | 55 | 19 | 8.21 |
| middle cingulate cortex | R | 2 | -9 | 35 | 2.48 |
|  | L | -2 | -13 | 33 | 3.09 |
| **temporal lobe** |  |  |  |  |  |
| superior temporal gyrus | R | 48 | 3 | -21 | 4.54 |
|  | R | 52 | -15 | -5 | 2.38 |
|  | L | -48 | -7 | 1 | 5.41 |
|  | R | 46 | 3 | -17 | 4.57 |
| superior temporal sulcus | R | 50 | -1 | -21 | 4.24 |
|  | R | 42 | -1 | 17 | 3.10 |
|  | L | -46 | -5 | -17 | -1.50 |
| middle temporal gyrus | R | 50 | -19 | -17 | 2.34 |
| **parietal lobe** |  |  |  |  |  |
| insula | L | -40 | 3 | -11 | 9.97 |
|  | R | 36 | 3 | -11 | 6.16 |
|  | R | 46 | 5 | -5 | 7.31 |
|  | R | 50 | 7 | 1 | 7.71 |
|  | R | 52 | 7 | 17 | 8.6 |
| **accumbens** | L | -2 | 17 | -11 | 2.08 |
|  | R | 2 | 27 | -11 | 2.42 |
| **putamen** | R | 26 | 15 | -5 | 4.49 |
| **caudate** | L | -14 | 13 | 3 | 1.74 |
|  | R | 16 | 15 | 3 | 2.71 |
|  | L | -6 | 13 | -5 | 2.26 |
|  | R | 6 | 15 | -5 | 1.18 |
|  | R | 20 | -1 | 23 | 2.54 |
| **thalamus** | L | -2 | -11 | 1 | 2.25 |
|  | R | 2 | -15 | -5 | 3.6 |
| **amygdala** | L | -26 | 1 | -25 | -8.54 |
|  | L | -22 | -1 | -21 | -6.57 |
|  | L | -30 | -11 | -17 | 3.90 |
| **parahipopocampal gyrus** | R | 40 | -17 | -21 | 2.49 |
| **hippocampus** | R | 50 | -3 | -23 | 3.22 |
