# Supplementary material for: Investigating the Predictive Value of Functional MRI to Appetitive and Aversive Stimuli: A Pattern Classification Approach
Source: PLoS One. 2016 Nov 21;11(11):e0165295. doi: 10.1371/journal.pone.0165295 (PMC5117589; doi:10.1371/journal.pone.0165295)
Supplement: S2 Table — Coordinates are shown in MNI, Wi: Highest weights within individual clusters. (DOCX) [file pone.0165295.s002.docx]

| **Region** | **Laterality** | **Coordinates** | | | **Wi** |
| --- | --- | --- | --- | --- | --- |
|  |  | **x** | **y** | **z** |  |
| **frontal lobe** | L | -4 | 47 | -15 | 2.73 |
| superior frontal gyrus | R | 2 | 49 | -15 | 2.25 |
|  | L | -2 | 55 | 23 | 6.54 |
|  | L | -2 | 47 | 47 | 10.04 |
|  | L | -4 | -5 | 65 | 10.29 |
|  | R | 4 | -7 | 65 | 4.49 |
|  | R | 2 | 45 | 23 | 6.15 |
| inferior frontal sulcus | L | -34 | 55 | 15 | 5.36 |
|  | R | 46 | 33 | 23 | 9.52 |
|  | L | -48 | 33 | 23 | 4.71 |
|  | R | 30 | 53 | 15 | 5.95 |
|  | L | -30 | -11 | 65 | 2.06 |
|  | R | 22 | 5 | 65 | 5.78 |
| inferior frontal gyrus | R | 56 | 15 | 7 | 7.08 |
|  | R | 50 | 9 | 7 | 7.83 |
|  | L | -40 | 39 | 15 | 6.68 |
|  | R | 42 | 37 | 15 | 6.34 |
| middle frontal gyrus | R | 36 | 57 | 7 | 5.51 |
|  | L | -34 | 51 | 7 | 2.95 |
|  | L | -32 | 51 | 23 | 5.51 |
|  | R | 38 | 43 | 23 | 7.29 |
|  | L | -32 | 43 | 29 | 4.87 |
| precentral gyrus | L | -58 | -7 | 33 | 11.44 |
|  | R | 54 | -1 | 33 | 9.7 |
| inferior precentral sulcus | L | -52 | -3 | 33 | 6.60 |
|  | L | -46 | 9 | 47 | 6.79 |
|  | R | 42 | 11 | 47 | 6.26 |
| lateral orbital gyrus | R | 46 | 41 | 7 | 6.00 |
| posterior orbital gyrus | L | -48 | 31 | 7 | 6.27 |
| subgenual ACC | L | -1 | 23 | -9 | -2 |
|  | R | 2 | 25 | -11 | 1.5 |
| pregenual ACC | R | 2 | 35 | 3 | 5.52 |
|  | L | -2 | 37 | 5 | 5.16 |
| posterior middle cingulate cortex | R | 2 | -11 | 41 | 6.07 |
|  | R | 2 | 13 | 31 | 2.55 |
|  | L | -2 | -11 | 29 | 1.71 |
|  | L | -2 | -11 | 45 | 9.5 |
|  | R | 1 | -5 | 47 | 9.7 |
|  | L | -2 | -11 | 37 | 6.38 |
| anterior middle cingulate cortex | R | 4 | 17 | 29 | 5.16 |
|  | L | -2 | 13 | 29 | 4.9 |
|  | L | -2 | 11 | 37 | 9.6 |
|  | R | 1 | 11 | 37 | 9.5 |
| **temporal lobe** |  |  |  |  |  |
| superior temporal gyrus | L | -40 | 11 | -22 | 5.05 |
|  | R | 50 | 5 | -15 | 2.9 |
|  | L | -50 | -5 | -13 | 2.15 |
|  | L | -56 | 3 | -5 | 12.3 |
| middle temporal gyrus | R | 48 | -9 | -23 | 2.20 |
| **parietal lobe** |  |  |  |  |  |
| insula | R | 34 | 9 | -15 | 6.29 |
|  | L | -42 | 3 | -15 | 7.54 |
|  | R | 46 | 15 | -5 | 11.38 |
|  | L | -44 | 5 | -5 | 8.14 |
| **putamen** | R | 16 | 9 | -5 | 6.60 |
|  | L | -24 | 7 | -5 | 5.0 |
|  | L | -32 | -11 | 7 | 3.4 |
|  | R | 20 | -1 | 7 | 4.52 |
| **caudate** | R | 8 | 13 | -5 | 3.73 |
|  | L | -10 | 13 | -5 | 3.24 |
|  | L | -10 | 3 | 7 | 6.7 |
|  | R | 10 | 3 | 7 | 6.75 |
| **amygdala** | R | 22 | -5 | -19 | 3.43 |
|  | L | -20 | 3 | -21 | 9.3 |
| **thalamus** | R | -10 | -17 | 7 | 7.47 |
|  | L | 8 | -23 | 7 | 6.70 |
