# Supplementary material for: Investigating the Predictive Value of Functional MRI to Appetitive and Aversive Stimuli: A Pattern Classification Approach
Source: PLoS One. 2016 Nov 21;11(11):e0165295. doi: 10.1371/journal.pone.0165295 (PMC5117589; doi:10.1371/journal.pone.0165295)
Supplement: S3 Table — Coordinates are shown in MNI, Wi: Highest weights within individual clusters. (DOCX) [file pone.0165295.s003.docx]

| **Region** | **Laterality** | **Coordinates** | | | **Wi** |
| --- | --- | --- | --- | --- | --- |
|  |  | **x** | **y** | **z** |  |
| **frontal lobe** |  |  |  |  |  |
| superior frontal gyrus | L | -2 | 47 | 47 | 11.69 |
|  | R | 2 | 47 | 47 | 11.55 |
|  | L | -14 | 59 | 21 | -1.97 |
| inferior frontal sulcus | L | -42 | 43 | 21 | 7.49 |
|  | R | 42 | 41 | 21 | 7.28 |
| inferior frontal gyrus | L | -48 | 23 | 21 | 8.35 |
|  | R | 46 | 25 | 21 | 6.45 |
|  | R | 52 | 7 | 9 | 6.2 |
|  | L | -56 | 11 | 9 | 7.52 |
| middle frontal gyrus | R | 46 | 33 | 27 | 11.71 |
|  | L | -36 | 47 | 27 | 6.13 |
|  | L | -26 | 55 | 15 | 5.2 |
|  | R | 30 | 53 | 15 | 6.52 |
| superior frontal sulcus | L | -22 | 11 | 47 | 2.22 |
|  | R | 24 | 9 | 47 | 3.12 |
| medial orbital gyrus | R | 16 | 33 | -19 | 1.6 |
| posterior orbital gyrus | L | -38 | 13 | -15 | 4.47 |
|  | R | 32 | 11 | -15 | 4.52 |
| subgenual ACC | L | -2 | 29 | -9 | -2.09 |
| pregenual ACC | R | 2 | 49 | 1 | 3.31 |
|  | L | -4 | 47 | 3 | 2.9 |
| middle cingulate cortex | L | -2 | 9 | 39 | 11.97 |
|  | R | 2 | 9 | 39 | 10.44 |
| **temporal lobe** |  |  |  |  |  |
| superior temporal gyrus | L | -60 | -7 | -5 | 16.16 |
|  | R | 58 | -5 | -5 | 6.66 |
|  | R | 56 | -15 | 5 | 7.30 |
|  | L | -42 | 13 | -23 | 1.5 |
| inferior precentral sulcus | L | -50 | 7 | 27 | 6.42 |
|  | R | 52 | 7 | 27 | 6.65 |
| **parietal lobe** |  |  |  |  |  |
| insula | L | -44 | 5 | -5 | 9.25 |
|  | R | 46 | 15 | -5 | 12.16 |
| **putamen** | L | -22 | 7 | -5 | 6.19 |
|  | R | 16 | 9 | -5 | 6.48 |
| **caudate** | L | -10 | 13 | 7 | 3.44 |
|  | R | 12 | 13 | 7 | 3.11 |
| **thalamus** | L | -4 | -19 | -15 | 3.59 |
|  | R | 6 | -17 | -15 | 3.15 |
| **amygdala** | L | -22 | 3 | -23 | 17.12 |
|  | R | 20 | -3 | -19 | 7.46 |
| **hippocampus** | L | -18 | 11 | -23 | 9.22 |
| **globo pallidus** | L | -8 | -13 | 7 | 7.12 |
|  | R | 2 | -19 | 7 | 6.9 |
