# Supplementary material for: Investigating the Predictive Value of Functional MRI to Appetitive and Aversive Stimuli: A Pattern Classification Approach
Source: PLoS One. 2016 Nov 21;11(11):e0165295. doi: 10.1371/journal.pone.0165295 (PMC5117589; doi:10.1371/journal.pone.0165295)
Supplement: S4 Table — Coordinates are shown in MNI, Wi: Highest weights within individual clusters. (DOCX) [file pone.0165295.s004.docx]

| **Region** | **Laterality** | **Coordinates** | | | **Wi** |
| --- | --- | --- | --- | --- | --- |
|  |  | **x** | **y** | **z** |  |
| **frontal lobe** | L | -2 | 43 | 3 | 4.9 |
| superior frontal gyrus | R | 8 | 47 | 3 | 4.22 |
|  | L | -16 | 55 | 23 | 5.12 |
|  | R | 24 | 49 | 23 | 4.9 |
|  | L | -10 | -9 | 53 | 8.65 |
|  | R | 10 | 35 | -11 | 3.75 |
|  | L | -2 | 41 | -11 | 4.18 |
| inferior frontal gyrus | R | 42 | 31 | 23 | 7.73 |
|  | R | 44 | 37 | 17 | 7.91 |
|  | L | -38 | 39 | 17 | 2.88 |
|  | R | 46 | 19 | 17 | 7.43 |
|  | L | -52 | 15 | 17 | 2.6 |
| middle frontal gyrus | R | 36 | 47 | 23 | 5.66 |
|  | L | -34 | 37 | 23 | 1.8 |
|  | L | -44 | 37 | 33 | -1.35 |
|  | R | 38 | 43 | 33 | -2.29 |
|  | L | -40 | 43 | 3 | 4.69 |
|  | R | 14 | 21 | 53 | 8.65 |
|  | L | -12 | 25 | 53 | 5.25 |
| inferior precentral sulcus | R | 54 | 5 | 23 | 9.25 |
| superior frontal sulcus | L | -30 | -3 | 57 | -1.37 |
|  | L | -22 | 17 | 53 | 3.86 |
| medial orbital gyrus | L | -6 | 35 | -21 | 4.03 |
|  | L | -6 | 33 | -21 | 3.62 |
|  | L | -4 | 35 | -17 | 3.06 |
|  | L | -18 | 31 | -17 | 4.93 |
| lateral orbital gyrus | L | -40 | 23 | -5 | 4.16 |
| posterior orbital gyrus | R | 42 | 13 | -11 | 7.30 |
| precentral gyrus | L | -54 | 31 | 7 | 4.15 |
|  | L | -54 | -5 | 23 | 4.54 |
|  | R | 52 | 1 | 33 | 9.40 |
| superior precentral sulcus | L | -42 | -1 | 53 | 2.56 |
|  | R | 40 | -1 | 53 | 5.6 |
| poscentral gyrus | R | 54 | -13 | 33 | 3.67 |
|  | L | -38 | -11 | 33 | 1.45 |
| pregenual anterior cingulate cortex | L | -4 | 35 | 13 | 7.9 |
|  | L | -4 | 57 | 21 | 7.8 |
|  | R | 2 | 35 | 13 | 8.58 |
|  | R | 2 | 55 | 19 | 8.21 |
| middle cingulate cortex | R | 2 | -9 | 35 | 2.48 |
|  | L | -2 | -13 | 33 | 3.09 |
| **temporal lobe** |  |  |  |  |  |
| superior temporal gyrus | R | 48 | 3 | -21 | 4.54 |
|  | R | 52 | -15 | -5 | 2.38 |
|  | L | -48 | -7 | 1 | 5.41 |
|  | R | 46 | 3 | -17 | 4.57 |
| superior temporal sulcus | R | 50 | -1 | -21 | 4.24 |
|  | R | 42 | -1 | 17 | 3.10 |
|  | L | -46 | -5 | -17 | -1.50 |
| middle temporal gyrus | R | 50 | -19 | -17 | 2.34 |
| **parietal lobe** |  |  |  |  |  |
| insula | L | -40 | 3 | -11 | 9.97 |
|  | R | 36 | 3 | -11 | 6.16 |
|  | R | 46 | 5 | -5 | 7.31 |
|  | R | 50 | 7 | 1 | 7.71 |
|  | R | 52 | 7 | 17 | 8.6 |
| **accumbens** | L | -2 | 17 | -11 | 2.08 |
|  | R | 2 | 27 | -11 | 2.42 |
| **putamen** | R | 26 | 15 | -5 | 4.49 |
| **caudate** | L | -14 | 13 | 3 | 1.74 |
|  | R | 16 | 15 | 3 | 2.71 |
|  | L | -6 | 13 | -5 | 2.26 |
|  | R | 6 | 15 | -5 | 1.18 |
|  | R | 20 | -1 | 23 | 2.54 |
| **thalamus** | L | -2 | -11 | 1 | 2.25 |
|  | R | 2 | -15 | -5 | 3.6 |
| **amygdala** | L | -26 | 1 | -25 | -8.54 |
|  | L | -22 | -1 | -21 | -6.57 |
|  | L | -30 | -11 | -17 | 3.90 |
| **parahipopocampal gyrus** | R | 40 | -17 | -21 | 2.49 |
| **hippocampus** | R | 50 | -3 | -23 | 3.22 |
